# Alzheimer's disease medication and outcomes of hospitalisation among patients with dementia

**DOI:** 10.1017/S2045796019000702

**Published:** 2019-11-14

**Authors:** T. Möllers, L. Perna, H. Stocker, P. Ihle, I. Schubert, B. Schöttker, L. Frölich, J. Bauer, H. Brenner

**Affiliations:** 1 Network Aging Research, Heidelberg University, Heidelberg, Germany; 2 Division of Clinical Epidemiology and Aging Research, German Cancer Research Center, Heidelberg, Germany; 3 Medical Faculty, Heidelberg University, Heidelberg, Germany; 4 Department of Translational Research in Psychiatry, Max Planck Institute of Psychiatry, Munich, Germany; 5 PMV Research Group, Faculty of Medicine and University Hospital Cologne, University of Cologne, Cologne, Germany; 6 Department of Gerontopsychiatry, Central Institute of Mental Health, Heidelberg University, Heidelberg, Germany; 7 Center for Geriatric Medicine, Heidelberg University, Agaplesion Bethanien Krankenhaus Heidelberg, Heidelberg, Germany

**Keywords:** Dementia, Alzheimer's disease, epidemiology, prospective study, psychopharmacology

## Abstract

**Aims:**

The use of Alzheimer disease medication for the treatment of dementia symptoms has shown significant benefits with regards to functional and cognitive outcomes as well as nursing home placement (NHP) and mortality. Hospitalisations in these patient groups are characterised by extended length of stays (LOS), frequent readmissions, frequent NHP and high-mortality rates. The impact of Alzheimer disease medication on the aforementioned outcomes remains still unknown. This study assessed the association of Alzheimer disease medication with outcomes of hospitalisation among patients with Alzheimer disease and other forms of dementia.

**Methods:**

A dynamic retrospective cohort study from 2004 to 2015 was conducted which claims data from a German health insurance company. People with dementia (PWD) were identified using ICD-10 codes and diagnostic measures. The main predictor of interest was the use of Alzheimer disease medication. Hospitalisation outcomes included LOS, readmissions, NHP and mortality during and after hospitalisation across four hospitalisations. Confounding was addressed using a propensity score throughout all analyses.

**Results:**

A total of 1380 users of Alzheimer disease medication and 6730 non-users were identified. The use of Alzheimer disease medication was associated with significantly shorter LOS during the first hospitalisations with estimates for the second, third and fourth showed a tendency towards shorter hospital stays. In addition, current users of Alzheimer disease medication had a lower risk of hospital readmission after the first two hospitalisations. These associations were not significant for the third and fourth hospitalisations. Post-hospitalisation NHP and mortality rates also tended to be lower among current users than among non-users but differences did not reach statistical significance.

**Conclusions:**

Our results indicate that Alzheimer disease medication might contribute to a reduction of the LOS and the number of readmissions in PWD.

## Introduction

The current pharmaceutical treatment options for Alzheimer disease are still limited to symptomatic interventions by cholinesterase inhibitors (ChEIs) and *N*-methyl-d-aspartate receptor (NMDA) antagonist memantine, which were both shown to improve functional and cognitive outcomes in a significant percentage of Alzheimer disease patients (Trinh *et al*., 2003; Howard *et al*., 2012; Zhu *et al*., 2013; Dou *et al*., 2018). Furthermore, studies on Alzheimer disease medication have also reported significant benefits for mortality (Zhu *et al*., 2013; Black *et al*., 2018; Mueller *et al*., 2018*a*; Pilotto *et al*., 2018) and a delay in nursing home placement (NHP) (Black *et al*., 2018; Wattmo *et al*., 2018) in this patient group. However, until now there have been only very few comprehensive epidemiologic studies investigating the association between the use of Alzheimer disease medications and hospitalisations.

Hospitalisations among people with any type of dementia are associated with severe adverse outcomes (Watkin *et al*., 2012), including functional decline (Pedone *et al*., 2005), a long length of stay (LOS) (Möllers *et al*., 2019a), high complication and mortality rates (Bail *et al*., 2013; Reynish *et al*., 2017; Lehmann *et al*., 2018; Pasina *et al*., 2018), a high rate of readmission (Pickens *et al*., 2017; Lehmann *et al*., 2018) and of NHP (Lehmann *et al*., 2018). Hence, the question whether the use of ChEIs and the NMDA antagonist memantine may limit the detrimental effects of long and complex hospitalisations for people with dementia (PWD) is of high public health relevance.

To date, only two studies have addressed the effects of Alzheimer disease medication on outcomes during hospitalisation among patients with Alzheimer disease. The first study showed an association of donepezil with reduced in-hospital mortality among Alzheimer disease patients with pneumonia (Abe *et al*., 2018). However, this study was not designed to evaluate the observed medication effects. The second study reported a reduced number of inpatient days for long-term treatment compared to short-term treatment with ChEIs, but it did not compare those two groups to non-users (Ku *et al*., 2018). Furthermore, neither study assessed the use of Alzheimer disease medication in patients with other forms of dementia or their association with outcomes after hospitalisation, even though Alzheimer disease medication is also used off-label among patients with vascular dementia, non-specific pathology or other dementia pathologies than Alzheimer disease or vascular dementia (Bohlken *et al*., 2015). This is of particular interest for patients with Lewy body dementia where ChEIs have shown effectiveness by improving cognitive function, behavioural disturbances and activities of daily living (Matsunaga *et al*., 2015; McKeith *et al*., 2017).

The aims of this study were to assess if PWD treated with Alzheimer disease medication have better outcomes of hospitalisation than PWD not treated with Alzheimer disease medication, including LOS, in-hospital mortality, hospital readmission, NHP and mortality after hospitalisation as well as to identify groups which might benefit the most from the use of Alzheimer disease medications.

## Methods

### Study design

A retrospective dynamic cohort study using claims data of German health insurers from 2004 to 2015 was conducted. The database itself consists of a statutory health insurance sample beginning in 1998 (18.75% random sample of all subjects insured by ‘Allgemeine Ortskrankenkasse (AOK) Hessen’) (Ihle *et al*., 2005). Patient informed consent was not required by law as the study was based on pseudonymous data. The utilisation of the database for research purposes was approved by the Ministry of Social Affairs of Hesse. Starting in 2006, cohort entry and cohort exit was possible in every year of the study period. The baseline period started 2 years prior to cohort entry to assess pre-existing comorbidities and medication use. Reasons for cohort exit were limited to death, end of insurance period or end of study period. End of insurance period relates to participants switching to a different statutory health insurance plan.

### Study population

A detailed description of the algorithm used to identify PWD has been previously provided elsewhere (Möllers *et al*., 2019b). In short, those eligible for cohort entry were all members of the statutory health insurance aged ⩾55 years during the study period with information on age and sex, and a continuous insurance period of at least 2 years prior to cohort entry. PWD were determined through two confirmed outpatient diagnoses in the same or consecutive 3-month periods or one inpatient diagnosis. Additionally, the application of at least one appropriate diagnostic measurement was required, which included testing of cerebrospinal fluid, computed tomography scan and magnetic resonance imaging of the head, or positron-emission tomography of the brain.

PWD diagnosis was assigned according to the type of dementia by using all dementia diagnoses in the first year after cohort entry. Since no specific ICD-10 code for mixed dementia exists, it was defined as the presence of Alzheimer disease and vascular dementia types according to ICD-10 coding. Repeat switching of specific dementia types or switching from a specific to an unspecific dementia type led to an assignment to other/unknown dementia.

### Outcomes

The first four hospitalisations after cohort entry with at least one overnight stay were considered independently to analyse the following outcomes: LOS, in-hospital mortality, hospital readmission, NHP and mortality after hospitalisation at 30 days, 60 days and 90 days after discharge from the hospital. In case the first dementia diagnosis was an inpatient diagnosis, this was considered the first hospitalisation. Overlapping periods of hospitalisation were considered as one hospitalisation period.

### Prescription of medications

Outpatient dispensation of the selected medication was assessed using the Anatomical Therapeutic Chemical Classification (ATC)/define daily dose (DDD) system. Participants were grouped into current users or non-users for each hospitalisation. Current users were defined as having a dispensation of Alzheimer disease medication which was dated before the hospitalisation and lasted at least until outcomes occurred or the outcome assessment ended (discharge from hospital for LOS and in-hospital mortality; 30/60/90 days after discharge for readmission, NHP and mortality) to ensure their exposure status throughout the outcome assessment. This was done by adding the package size in form of the DDD to the date of dispensation from the pharmacy (e.g. 100 DDDs last for 100 days). Hospitalised patients are provided with the necessary medication from the hospital and do not have to use the medications provided by the outpatient pharmacy. To account for this, the LOS of the hospitalisation was added to the date of dispensation and DDDs (e.g. package size of 100 days + LOS of 15 days lasts for 115 days). Alzheimer disease medication used in this study included ChEIs (donepezil, galantamine and rivastigmine) and memantine.

### Propensity score

To accurately assess the effects of Alzheimer disease medication on the outcomes by minimising bias due to confounding high-dimensional propensity scores were constructed (Schneeweiss *et al*., 2009). For comparing current users and non-users of Alzheimer disease medication, the propensity scores included are age at baseline, sex, type of dementia, time since dementia diagnosis, need for care as a proxy for the severity of dementia (reflects the ability to perform activities of daily living, dependency and utilisation of care), as well as the top 150 comorbidities (third level ICD-10 codes, e.g. F32 for depressive episode) and top 150 medications (five digit ATC codes, e.g. N06AB for selective serotonin reuptake inhibitors) based on their potential to control confounding. For stratified analysis, the propensity scores only included variables not used for stratification. Since care dependency, severity and impact of comorbidities and number of medications can change over the course of the disease (Solomon *et al*., 2011; Schüssler and Lohrmann, 2015; Haaksma *et al*., 2017; Gnjidic *et al*., 2018) the propensity score and the included variables were reassessed for the grouping into current users and non-users before each of the hospitalisations. Although the last treatment decision before hospitalisation would be the optimal reassessment point, non-users do not have a dispensation date available. Hence, the assessment was done from the baseline period until the 3-month period prior to each hospitalisation to have the same assessment points for current users and non-users. The time of the last treatment decision for current users was almost exclusively (99%) in the 3-month period before hospitalisation (online Supplementary Table S1).

### Statistical analysis

For the purpose of descriptive statistics the study participants were grouped into those who used Alzheimer disease medication at some point in the follow-up and those who never used Alzheimer disease medication. Differences of study characteristics were investigated with *χ*^2^ test for categorical variables, *t*-tests or (in case of violations of the normal distribution assumption) Wilcoxon–Mann–Whitney tests for continuous variables, and Kruskal–Wallis tests for median values with a level of statistical significance of *p* < 0.05. The association of Alzheimer disease medication with LOS was examined using multivariate linear regression for comparability to other studies analysing LOS (Möllers *et al*., 2019a). Additionally the LOS was analysed using Poisson regression to display rate ratios of the mean inpatient days. Associations between Alzheimer disease medication and all remaining outcomes were analysed using Cox regression. Analyses were adjusted using the respective propensity scores as well as year of hospitalisation, the main discharge diagnosis and exposure time to Alzheimer disease medication. Stratified analyses were conducted to explore whether or not certain patient groups benefited more from Alzheimer disease medication than others as it is known from previous studies that for example age and type of dementia play a role in LOS (Mueller *et al*., 2018*b*; Möllers *et al*., 2019b). All statistical analyses were performed using SAS 9.4 (SAS Institute Inc., Cary, NC, USA).

### Sensitivity analysis

The following sensitivity analyses were conducted. First, participants who deceased during hospitalisation were excluded to compare the LOS between current users and non-users, because participants who deceased during hospitalisation might have shortened hospital stays. Second, participants who deceased after hospitalisation within the specific time limits (30, 60 and 90 days) but after readmission or NHP were excluded to compare the risk of readmission and NHP of current users and non-users, because deceased participants might have a higher risk to be readmitted to the hospital or were transferred to a nursing home before their death. Furthermore competing risk analyses were conducted for NHP and readmission using Cox regression.

## Results

### Sample characteristics

Summary statistics comparing Alzheimer disease medication users and non-users are shown in Table 1. The study population consisted of 1380 users and 6730 non-users. Approximately 60% in both groups were female. Non-users of Alzheimer disease medication were on average about 1 year older than users (79.75 years *v.* 78.13 years). Furthermore, the proportions of patients with vascular dementia and other/unknown dementia types were higher among non-users than among users. At cohort entry, non-users more often had cardiovascular and pulmonary diseases, as well as diabetes than users. In contrast, prevalent depression and bone/joint diseases were more common among users.

**Table 1.** Study characteristics of Alzheimer's disease medication users and non-users

	User (*N* = 1380)	Non-user (*N* = 6730)	*p*-Value^a^
Demographic and hospitalisation characteristics			
Sex (female, *N*, %)	832 (60.3)	4115 (61.1)	0.560
Age in years at baseline (mean, s.d.)	78.1 (6.8)	79.8 (7.9)	<0.001
Years of follow-up (median, IQR)	3.0 (3.6)	2.0 (3.2)	<0.001
Reasons for leaving study (*N*, %)			
Deceased	820 (59.4)	4051 (60.2)	0.610
End of study period	529 (38.3)	2553 (37.9)	
End of insurance period	31 (2.3)	126 (1.9)	
Type of dementia (*N*, %)			
Alzheimer's dementia	419 (30.4)	927 (13.8)	<0.001
Vascular dementia	288 (20.9)	2155 (32.0)	
Other, unknown	639 (46.3)	3517 (52.3)	
Lewy body dementia	34 (2.5)	131 (2.0)	
Number of hospitalisations (median, IQR)	3.0 (3.0)	3.0 (4.0)	<0.001
LOS first hospitalisation in days (mean, s.d.)^b^	14.1 (15.4)	18.1 (17.7)	<0.001
LOS second hospitalisation in days (mean, s.d.)^c^	12.9 (12.5)	14.2 (13.8)	0.004
LOS third hospitalisation in days (mean, s.d.)^d^	12.2 (13.4)	12.8 (13.5)	0.042
LOS fourth hospitalisation in days (mean, s.d.)^e^	10.8 (10.3)	11.9 (12.5)	0.224
Comorbidities at cohort entry (*N*, %)			
Cardiovascular diseases	646 (46.8)	4033 (59.9)	<0.001
Neurologic diseases	219 (15.9)	1216 (18.1)	0.056
Depression	511 (37.0)	2319 (34.5)	0.062
Pulmonary diseases	240 (17.4)	1350 (20.1)	0.026
Diabetes	442 (32.0)	2461 (36.6)	0.001
Bone/joint diseases	848 (61.5)	3913 (58.1)	0.024
Cancer	259 (18.8)	1404 (20.9)	0.077
Other comorbidities^f^	270 (19.6)	1944 (28.9)	<0.001

For comparison of baseline characteristics the study participants were grouped into those who used Alzheimer dementia medication at some point during the follow-up and those who never used Alzheimer dementia medication.

a *p*-values derived from the *t*-test or Wilcoxon–Mann–Whitney tests for continuous and *χ*^2^ tests for categorical variables.

b Current users: 749; non-users: 6864.

c Current users: 774; non-users: 5253.

d Current users: 528; non-users: 3529.

e Current users: 328; non-users: 2437.

f Chronic kidney disease, alcohol abuse, dehydration.

### Outcomes

Propensity score adjusted outcomes of LOS and in-hospital mortality are reported in Table 2. Current users of Alzheimer disease medication had a significantly shorter first [−4.54 days, 95% confidence interval (CI): −5.82, −3.25] hospitalisation compared to non-users. Although the estimates tended to show a reduction in LOS for the second, third and fourth hospitalisations, no significant differences were present in linear regression models. Rate ratios of the mean inpatient days showed significant shorter stays of 25 to 6% for the first, second and third hospitalisations of current users. There were no significant differences regarding in-hospital mortality between current users and non-users during any of the four hospitalisations.

**Table 2.** Propensity score adjusted estimates of mean difference in days (MD) and rate ratios (RR) for LOS and of HRs for in-hospital mortality (non-users as the reference group)

	LOS		
Hospitalisation	MD (95% CI)	RR (95% CI)	In-hospital mortality HR (95% CI)
First^a^	−4.54 (−5.82; −3.25)	0.75 (0.74; 0.77)	1.03 (0.62; 1.70)
Second^b^	−0.85 (−1.77; 0.08)	0.94 (0.92; 0.96)	0.85 (0.60; 1.21)
Third^c^	−0.76 (−1.94; 0.42)	0.94 (0.91; 0.97)	0.72 (0.47; 1.12)
Fourth^d^	−0.10 (−1.54; 1.34)	0.99 (0.95; 1.03)	1.19 (0.73; 1.93)

Multivariate linear and Poisson regression for LOS and Cox regression for in-hospital mortality.

Additional adjustment with year of hospitalisation, the main discharge diagnosis and exposure time.

a Current users: 749; non-users: 6864.

b Current users: 774; non-users: 5253.

c Current users: 528; non-users: 3529.

d Current users: 328; non-users: 2437.

The propensity score adjusted outcomes within various time windows for readmission, NHP and mortality after hospitalisation are presented in Table 3. PWD currently receiving Alzheimer disease medication had a significantly lower risk to be readmitted after the first and second hospitalisations. Current users had a significantly reduced risk to be readmitted after 30 days [hazard ratio (HR): first hospitalisation: 0.72, 95% CI: 0.59, 0.87; second hospitalisation: 0.73, 95% CI: 0.59, 0.89], 60 days (HR first hospitalisation: 0.71, 95% CI: 0.60, 0.83; second hospitalisation: 0.80, 95% CI: 0.68, 0.94) and 90 days (HR first hospitalisation: 0.74, 95% CI: 0.64, 0.85; second hospitalisation: 0.80, 95% CI: 0.69, 0.93) compared to non-users. The estimates for the third and fourth hospitalisations showed a tendency for a reduced risk of readmission but did not reach statistical significance. Current users also tended to have lower NHP and mortality after hospitalisations. However, only results for NHP reached statistical significance after the third hospitalisation only and the results for mortality only reached statistical significance within 60 days after the first hospitalisation.

**Table 3.** Propensity score adjusted estimates for readmission, NHP and mortality within defined time windows after hospitalisation displayed as HRs (non-users as the reference group)

Hospitalisation	Readmission HR (95% CI)	NHP HR (95% CI)	Mortality HR (95% CI)
First^a^			
30 days	0.72 (0.59; 0.87)	0.86 (0.70; 1.04)	0.75 (0.45; 1.23)
60 days	0.71 (0.60; 0.83)	0.86 (0.71; 1.05)	0.67 (0.46; 0.97)
90 days	0.74 (0.64; 0.85)	0.85 (0.70; 1.04)	0.80 (0.59; 1.09)
Second^b^			
30 days	0.73 (0.59; 0.89)	0.91 (0.77; 1.08)	0.93 (0.64; 1.34)
60 days	0.80 (0.68; 0.94)	0.91 (0.77; 1.08)	0.86 (0.64; 1.16)
90 days	0.80 (0.69; 0.93)	0.91 (0.77; 1.08)	0.83 (0.63; 1.08)
Third^c^			
30 days	0.86 (0.67; 1.10)	0.80 (0.66; 0.97)	1.11 (0.73; 1.67)
60 days	0.85 (0.69; 1.04)	0.80 (0.66; 0.96)	1.00 (0.71; 1.40)
90 days	0.81 (0.67; 0.98)	0.80 (0.66; 0.97)	1.01 (0.75; 1.35)
Fourth^d^			
30 days	0.87 (0.63; 1.20)	0.98 (0.77; 1.23)	0.88 (0.45; 1.69)
60 days	0.87 (0.66; 1.14)	0.98 (0.77; 1.23)	0.99 (0.60; 1.62)
90 days	0.90 (0.70; 1.15)	0.98 (0.77; 1.23)	1.36 (0.93; 1.99)

Cox regression for all three outcomes.

Additional adjustment with year of hospitalisation, the main discharge diagnosis and exposure time.

a 30 days: current users: 731; non-users: 6593; 60 days: current users: 713, non-users: 6332; 90 days: current users: 681, non-users: 5828.

b 30 days: current users: 735, non-users: 4639; 60 days: current users: 700, non-users: 4408; 90 days: current users: 648, non-users: 3939.

c 30 days: current users: 504, non-users: 3252; 60 days: current users: 473, non-users: 3044; 90 days: current users: 429, non-users: 2701.

d 30 days: current users: 305, non-users: 2259; 60 days: current users: 293, non-users: 2144; 90 days: current users: 272, non-users: 1933.

### Stratified analyses

Stratified estimates for LOS by patient subgroups are shown in Table 4 and Table S2. In the stratified analyses, significant differences between current users and non-users were mainly seen for the first hospitalisation. In age-specific analyses, reduction of LOS of the first hospitalisation was particularly strong among 55–64 year old patients (−5.83, 95% CI: −10.36, −1.29). No major differences were seen by sex. Significant reductions were seen for Alzheimer disease (−2.22, 95% CI: −3.83; −0.61) and other/unknown types of dementia (−1.60, 95% CI: −2.76; −0.43) for the first hospitalisation. Estimates for vascular dementia and Lewy body dementia showed a reduction but were not statistically significant. Reduction of LOS of the first and second hospitalisations appeared to be strongest within the first three months following diagnosis. The results of the stratified analysis regarding the remaining outcomes are not displayed here as no specific trends could be observed.

**Table 4.** Stratified estimates for LOS as mean difference in days (MD) (non-users as the reference group)

	First hospitalisation^a^	Second hospitalisation^b^	Third hospitalisation^c^	Fourth hospitalisation^d^
Hospitalisation	LOS MD (95% CI)	LOS MD (95% CI)	LOS MD (95% CI)	LOS MD (95% CI)
Age group (in years) at baseline				
55–64	−5.83 (−10.36; −1.29)	−3.31 (−7.30; 0.67)	−0.44 (−5.31; 4.43)	5.51 (−2.29; 13.31)
65–74	−1.03 (−2.69; 0.62)	−0.18 (−1.64; 1.30)	−2.61 (−4.45; −0.77)	0.76 (−1.53; 3.05)
75–84	−2.00 (−3.09; −0.89)	−0.75 (−1.73; 0.22)	−0.16 (−1.36; 1.04)	0.07 (−1.41; 1.56)
85–94	−2.10 (−4.12; −0.09)	−0.62 (−2.38; 1.13)	−0.18 (−2.27; 1.91)	−0.98 (−3.55; 1.59)
95+	−0.72 (−10.67; 9.23)	−3.17 (−10.06; 3.71)	−2.92 (−27.96; 22.12)	–
Sex				
Female	−1.46 (−2.51; −0.41)	−0.83 (−1.79; 0.12)	−0.38 (−1.14; 0.78)	0.47 (−0.98; 1.93)
Male	−2.70 (−3.98; −1.40)	−0.66 (−1.79; 0.47)	−1.14 (−2.50; 0.22)	0.02 (−1.67; 1.71)
Type of dementia				
AD	−2.22 (−3.83; −0.61)	−0.22 (−1.61; 1.17)	−0.47 (−2.23; 1.28)	−0.51 (−2.58; 1.56)
Vascular dementia	−1.64 (−3.35; 0.08)	−0.17 (−1.79; 1.44)	−0.50 (−2.49; 1.49)	1.47 (−0.78; 3.71)
Lewy body dementia	−2.88 (−8.76; 3.00)	1.60 (−4.02; 7.23)	−3.17 (−8.02; 1.67)	0.40 (−7.10; 7.85)
Other, unknown type	−1.60 (−2.76; −0.43)	−1.33 (−2.37; −0.29)	−0.73 (−1.98; 0.52)	−0.10 (−1.74; 1.54)
Time since diagnosis per hospitalisation (in months)				
First tertile	0–3	0–3	0–9	0–15
	−2.95 (−4.23; −1.67)	−2.46 (−4.53; −0.38)	−1.69 (−3.71; 0.34)	1.21 (−0.84; 3.26)
Second tertile	>3	4–12	10–24	16–30
	−0.26 (−1.30; 0.78)	0.16 (−1.01; 1.33)	0.04 (−1.43; 1.51)	−0.43 (−2.67; 1.80)
Third tertile		>12	>24	>30
	–	−0.63 (−1.67; 0.41)	−0.73 (−2.03; 0.56)	0.15 (−1.48; 1.78)

Multivariate linear regression for LOS.

Adjusted by the propensity score with the exception of the stratified variable as well as year of hospitalisation, the main discharge diagnosis and exposure time.

a Current users: 749; non-users: 6864.

b Current users: 774; non-users: 5253.

c Current users: 528; non-users: 3529.

d Current users: 328; non-users: 2437.

### Sensitivity analyses

Sensitivity analyses for the LOS, risk of readmission and NHP are shown in online Supplementary Tables S3 and S4, respectively. Excluding participants who deceased during hospitalisation did not alter the previous reported results significantly as the first hospitalisation still showed a significant reduction for current users compared to non-users (−4.33, 95% CI: −5.06; −3.06) and the second and third hospitalisations tended to show a reduced LOS. Furthermore, rate ratios for the first, second and third hospitalisations still showed a significantly reduced LOS of 24 to 5% of current users.

When excluding participants who deceased within 30, 60 and 90 days after discharge from the hospital but after readmission or NHP, the estimates for the risk of readmission remained similar, showing a significantly reduced risk of readmission for current users compared to non-users after the first two hospitalisations. Estimates for the third and fourth hospitalisations tended to show a risk reduction for current users. In addition, the estimates for NHP remained fairly similar as well with a significant risk reduction after the third hospitalisation. The estimates for competing risk analyses were nearly identical for readmission and NHP as compared to the analyses excluding deceased patients.

## Discussion

In this comprehensive study with a large sample size, the association between Alzheimer disease medication and outcomes of hospitalisation among PWD was analysed. The current use of Alzheimer disease medication was associated with a temporal and significant reduction of LOS and the risk of readmission. This pattern was particularly pronounced for younger patients and within the first 3 months after diagnosis.

Only one other study conducted in Taiwan and partially comparable to the current study addressed the relationship between LOS and Alzheimer disease medication and found less inpatient days among long-term Alzheimer disease medication users compared to short-term users (Ku *et al*., 2018). Possible reasons explaining the reduced LOS and readmission after the first and second hospitalisations among the current users of Alzheimer disease medication might include the beneficial effects of Alzheimer disease medication on cognitive and functional outcomes (Trinh *et al*., 2003; Howard *et al*., 2012; Zhu *et al*., 2013; Dou *et al*., 2018), including the amelioration of neuropsychiatric symptoms (Gauthier *et al*., 2008; Wilcock *et al*., 2008; Kavanagh *et al*., 2011) and possibly the reduction of the incidence of delirium (Dautzenberg *et al*., 2004; Youn *et al*., 2017). Neuropsychiatric symptoms and delirium could have led otherwise to lower quality of life (Ready *et al*., 2004; Hurt *et al*., 2008; Conde-Sala *et al*., 2016), longer LOS (Möllers *et al*., 2019b) and worse cognitive and functional outcomes among PWD (Poulin *et al*., 2017). Thus improving cognitive, functional and neuropsychiatric outcomes as well as possibly reducing the incidence of delirium by the use of Alzheimer disease medication might lead to shorter stays and fewer readmissions. However, the associations of Alzheimer disease medication with LOS and readmission were not significant for the third and fourth hospitalisations, which might have been caused by reduced effects of Alzheimer disease medication over the course of the disease and the deterioration of the overall function, the latter being influenced among other factors by the multiple hospital admissions themselves. This is supported by our stratified analysis showing significant reductions of LOS among current users diagnosed 0–3 months prior to the first and second hospitalisations compared to non-users, and non-significant differences for the third and fourth hospitalisations. This absence of significant differences later during the disease might be also related to milder side effects of Alzheimer disease medication, among others pneumonia, bradycardia and syncope which might have led to hospitalisation (Gill *et al*., 2009; Park-Wyllie *et al*., 2009; Kim *et al*., 2011; Lampela *et al*., 2017). However, it has recently been shown that the risk for severe cardiac events including stroke, acute myocardial infarction and acute coronary syndrome is not increased among ChEI users (Isik *et al*., 2018).

Particularly strong associations in subgroups for the LOS of the first hospitalisation were shown regarding age, Alzheimer disease patients or patients with other/unknown types of dementia as well as PWD diagnosed 0–3 months before the first and second hospitalisations. In particular, the 55–64 year olds showed significant shorter stays of 5 days. A possible explanation could be that younger patients currently using Alzheimer disease medication might deteriorate slower with regards to functional and cognitive capacities or they may have been at a less severe stage of the disease, which can result in shorter hospital stays (Zhu *et al*., 2015). However, this would need to be investigated by future studies in detail. As expected, patients with Alzheimer disease benefit more from Alzheimer disease medication regarding LOS than patients with any other type of dementia. Nevertheless, it is interesting to note that current users in the group of other/unknown dementia also had a significant shorter first hospitalisation than non-users. One possible explanation is that the group other/unknown dementia in our cohort might include a high percentage of mixed dementia cases as well as uncertain diagnosis of Alzheimer disease and Lewy body dementia. Lewy body dementia is often un- or misdiagnosed but its management with ChEIs has shown effectiveness in improving cognition, global function and activities of daily living (Vann Jones and O'Brien, 2014; Matsunaga *et al*., 2015; McKeith *et al*., 2017). Treatment of mixed dementia with Alzheimer disease medication would also be in line with German national guidelines (German Society for Psychiatry *et al*., 2016). Another important finding of this study is the difference in LOS among current users and non-users with regards to time since cohort entry. This observation gives reason to believe that newly diagnosed PWD have greater benefits in terms of LOS when using Alzheimer disease medication.

Even though current Alzheimer disease medication use also tended to be associated with lower rates of NHP, in-hospital mortality and mortality after hospitalisation, with the exception of NHP after the third hospitalisation these associations did not reach statistical significance. Although some studies have shown positive effects of Alzheimer disease medication on NHP and mortality (Zhu *et al*., 2013; Black *et al*., 2018; Mueller *et al*., 2018*a*; Pilotto *et al*., 2018; Wattmo *et al*., 2018) none of those studies assessed these outcomes specifically after hospitalisation. A study among PWD hospitalised for pneumonia reported lower in-hospital mortality for PWD receiving donepezil, a ChEI. However, this study was not designed to evaluate the effects of Alzheimer disease medication on mortality (Abe *et al*., 2018).

In conclusion, our data suggest that Alzheimer disease medication, in addition to treating dementia symptoms, may also contribute to the reduction of negative effects of hospitalisation on a short-term basis, specifically LOS and readmissions. This can be interpreted as a positive ‘side-effect’ of Alzheimer disease medication. Nevertheless the overall negative effects of hospitalisations in PWD have to be addressed by multicomponent interventions, including early discharge planning (Fox *et al*., 2013), the use of a targeted care bundle (Koehler *et al*., 2009), the transitional care model (Naylor *et al*., 2014), special care units (Chiu *et al*., 2009; Spencer *et al*., 2013; Anderson *et al*., 2016), person-centred care (Tay *et al*., 2018), family-care including a comprehensive support programme for caregivers (Eloniemi-Sulkava *et al*., 2009) and improving caregiver well-being (Mittelman *et al*., 2006).

### Strength and limitations

Our study has several limitations. The analysed data originate from a single German region, which limits the generalisability of our results. Additionally, the analysis relied on ICD-10-GM codes for billing purposes in the SHI-System, which should ensure a high degree of validity but was not externally validated. However, we tried to ensure a valid diagnosis of dementia, as well as the underlying aetiology by using diagnostic measurements as part of our case definition and all diagnoses made in the first year after cohort entry. In accordance with drug registration and indication, Alzheimer disease medication users had a higher percentage of Alzheimer disease diagnoses, while non-users had more diagnoses of vascular dementia. Whether this fully reflects off-label use of Alzheimer disease medication in vascular dementia or misdiagnosis cannot be determined. At any rate, this may have led to an underestimation of the actual impact of Alzheimer disease medication on outcomes, even if many cases of dementia possess shared Alzheimer and vascular pathology (Attems and Jellinger, 2014; Jellinger and Attems, 2015). Since there was no information on the severity of dementia available, the need for care (reflects the ability to perform activities of daily living, dependency and utilisation of care) was used as a proxy variable. Although utilisation of and dependency on care increases over time and with the severity of dementia (Schüssler and Lohrmann, 2015; Michalowsky *et al*., 2016) and propensity scores were constructed to adjust for factors influencing the treatment decision and the outcomes of interest this might have left some residual confounding by indication. It is to be noted that the means of the propensity scores of non-users and current users (first: 0.36 *v.* 0.40; second: 0.37 *v.* 0.39; third: 0.36 *v.* 0.39; fourth: 0.35 *v.* 0.40) as well as the ranges within each group (first: 0.11–0.96; second: 0.14–0.96; third: 0.21–0.94; fourth: 0.21–0.95) were comparable. Furthermore, information on nutritional status, education, clinical parameters and family support was not available. Immortal time bias is not an issue in our study as we neither included a fixed endpoint for exposure assessment after cohort entry nor was cohort entry based on the prescription of Alzheimer disease medication. Rather, we assessed the first four hospitalisations after cohort entry and then grouped participants into current users and non-users for each hospitalisation based on the provided definitions to ensure their exposure status during outcome assessment. Furthermore, the exposure time was included in the analyses. Hence, previously unexposed time was counted as such and not as exposed time.

Among the strengths of our study is the novel assessment of the relationship between Alzheimer disease medication and hospital outcomes. This includes the findings of a temporarily reduced LOS and risk of readmission among current Alzheimer disease medication users. Further strengths of our study include a large sample size, the differentiation between multiple hospitalisations and the inclusion of patients independently of their living situation, health status or nationality. Moreover, using the comprehensive propensity score methodology enabled a more reliable conclusion on the treatment effects of Alzheimer disease medication on outcomes of hospitalisation. Finally, based on the nature of the data, recall or interviewer bias was avoided.

## Conclusion

Alzheimer disease medication may contribute to a temporary reduction of LOS and readmissions in PWD, especially in those at a younger age when diagnosed with Alzheimer disease and also shortly after the primary diagnosis of dementia. Potential long-term effects on LOS and readmissions as well as benefits regarding NHP and mortality after hospitalisation could not be established with certainty and should be investigated in the future, ideally in much larger studies.

## Supplementary material

For supplementary material accompanying this paper visit http://dx.doi.org/10.1017/S2045796019000702. click here to view supplementary material

## Data

The data used in this study cannot be shared as it was based on claims data of a German health insurance company, maintained by the PMV research group, and can only be used in the offices of the PMV research group.
