# Supplementary material for: Alzheimer's disease medication and outcomes of hospitalisation among patients with dementia
Source: Epidemiol Psychiatr Sci. 2019 Nov 14;29:e73. doi: 10.1017/S2045796019000702 (PMC8060969; doi:10.1017/S2045796019000702)
Supplement: Supplementary file 1 [file S2045796019000702sup001.docx]

**Supplementary material**

Table S1. Distribution of the last treatment decision of users before each hospitalization.

|  | 3-month period before, n (%) | Earlier than 3-month period, n (%) |
| --- | --- | --- |
| First hospitalization | 745 (99.47%) | 4 (0.53%) |
| Second hospitalization | 766 (98.97%) | 8 (1.03%) |
| Third hospitalization | 527 (99.81%) | 1 (0.19%) |
| Fourth hospitalization | 325 (99.09%) | 3 (0.91%) |

Table S2. Stratified estimates for Length of stay (LOS) as rate ratios (RR) (non-users as the reference group).

| Hospitalization | First hospitalization^a^ | Second hospitalization^b^ | Third hospitalization^c^ | Fourth hospitalization^d^ |
| --- | --- | --- | --- | --- |
|  | LOS RR (95% CI) | LOS RR (95% CI) | LOS RR (95% CI) | LOS MD (95% CI) |
| Age group (in years) at baseline |  |  |  |  |
| 55 to 64 | 0.59 (0.52; 0.69) | 0.72 (0.63; 0.82) | 0.96 (0.80; 1.14) | 1.52 (1.22; 1.90) |
| 65 to 74 | 0.92 (0.88; 0.96) | 0.99 (0.94; 1.03) | 0.78 (0.74; 0.84) | 1.08 (0.99; 1.17) |
| 75 to 84 | 0.86 (0.83; 0.89) | 0.94 (0.91; 0.97) | 0.99 (0.95; 1.03) | 1.01 (0.95; 1.06) |
| 85 to 94 | 0.85 (0.80; 0.90) | 0.95 (0.89; 1.01) | 0.98 (0.91; 1.07) | 0.90 (0.80; 1.01) |
| +95 | 0.93 (0.67; 1.29) | 0.74 (0.53; 1.02) | 0.90 (0.28; 2.91) | - |
| Sex |  |  |  |  |
| female | 0.90 (0.87; 0.92) | 0.93 (0.91; 0.96) | 0.96 (0.93; 1.00) | 1.05 (0.99; 1.11) |
| male | 0.80 (0.77; 0.83) | 0.95 (0.91; 0.98) | 0.90 (0.85; 0.94) | 1.00 (0.94; 1.07) |
| Type of dementia |  |  | | |
| AD | 0.84 (0.81; 0.88) | 0.98 (0.94; 1.03) | 0.96 (0.90; 1.02) | 0.95 (0.87; 1.03) |
| Vascular dementia | 0.89 (0.85; 0.93) | 0.99 (0.94; 1.04) | 0.96 (0.89; 1.02) | 1.14 (1.05; 1.24) |
| Lewy Body Dementia | 0.83 (0.70; 0.97) | 1.13 (0.98; 1.31) | 0.76 (0.63; 0.92) | 1.20 (0.96; 1.51) |
| Other, unknown Type | 0.88 (0.85; 0.91) | 0.89 (0.86; 0.92) | 0.93 (0.89; 0.98) | 0.99 (0.93; 1.06) |
| Time since diagnosis per hospitalization (in months) |  |  |  |  |
| First tertile | 0.84 (0.81; 0.88) | 0-3 | 0-9 | 0-15 |
|  | 0.89 (0.85; 0.93) | 0.82 (0.77; 0.88) | 0.86 (0.81; 0.92) | 1.13 (1.04; 1.22) |
|  | 0.83 (0.70; 0.97) |  |  |  |
| Second tertile | 0.88 (0.85; 0.91) | 4-12 | 10-24 | 16-30 |
|  |  | 1.02 (0.98; 1.06) | 1.00 (0.95; 1.06) | 0.95 (0.88; 1.04) |
|  | 0-3 |  |  |  |
| Third tertile | 0.80 (0.78; 0.83) | >12 | >24 | >30 |
|  |  | 0.95 (0.91; 0.98) | 0.93 (0.88; 0.98) | 1.02 (0.95; 1.08) |
| Poisson regression for LOS. | | | | |
| Adjusted by the propensity score with the exception of the stratified variable as well as year of hospitalization, the main discharge diagnosis, and exposure time. | | | | |
| a) User: 749; Non-user: 6,864. | | | | |
| b) User: 774; Non-user: 5,253. | | | | |
| c) User: 528; Non-user: 3,529. | | | | |
| d) User: 328; Non-user: 2,437. | | | | |

Table S3. Propensity score adjusted estimates of mean difference in days (MD) and rate ratios (RR) for length of stay (LOS) without participants deceased during hospitalization (non-users as the reference group).

| Hospitalization | LOS | |
| --- | --- | --- |
|  | MD (95% CI) | RR (95% CI) |
| First^a^ | -4.33 (-5.06; -3.06) | 0.76 (0.74; 0.78) |
|  |  |  |
| Second^b^ | -0.86 (-1.79; 0.08) | 0.94 (0.91; 0.96) |
|  |  |  |
| Third^c^ | -0.66 (-1.88; 0.56) | 0.95 (0.92; 0.98) |
|  |  |  |
| Fourth^d^ | 0.24 (-1.27; 1.76) | 1.02 (0.98; 1.07) |
| Multivariate linear regression and Poisson regression for LOS. | | |
| Additional adjustment with year of hospitalization, the main discharge diagnosis, and exposure time. | | |
| a) User: 731; Non-user: 6,593. | | |
| b) User: 735; Non-user: 4,639. | | |
| c) User: 504; Non-user: 3,252. | | |
| d) User: 305; Non-user: 2,259. | | |

Table S4. Propensity score adjusted estimates for Readmission and Nursing home placement (NHP) within defined time windows after hospitalization displayed as hazard ratios (HR) without deceased participants (exclusion) and competing risk analyses for mortality (non-users as the reference group).

| Hospitalization | Readmission | | NHP | |
| --- | --- | --- | --- | --- |
|  | Exclusion  HR (95% CI) | Competing risk  HR (95% CI) | Exclusion  HR (95% CI) | Competing risk HR (95% CI) |
| First^a^ |  |  |  |  |
| 30 days | 0.72 (0.59; 0.88) | 0.73 (0.60; 0.89) | 0.88 (0.73; 1.08) | 0.89 (0.73; 1.09) |
| 60 days | 0.73 (0.62; 0.88) | 0.75 (0.63; 0.89) | 0.91 (0.75; 1.11) | 0.93 (0.76; 1.13) |
| 90 days | 0.76 (0.65; 0.89) | 0.77 (0.66; 0.90) | 0.89 (0.72; 1.08) | 0.90 (0.74; 1.10) |
| Second^b^ |  |  |  |  |
| 30 days | 0.71 (0.58; 0.88) | 0.72 (0.58; 0.89) | 0.91 (0.76; 1.08) | 0.91 (0.77; 1.08) |
| 60 days | 0.80 (0.68; 0.95) | 0.82 (0.69; 0.97) | 0.94 (0.79; 1.12) | 0.96 (0.80; 1.14) |
| 90 days | 0.83 (0.71; 0.97) | 0.86 (0.74; 1.01) | 0.91 (0.77; 1.09) | 0.94 (0.79; 1.13) |
| Third^c^ |  |  |  |  |
| 30 days | 0.87 (0.67; 1.14) | 0.87 (0.67; 1.14) | 0.82 (0.68; 0.99) | 0.82 (0.68; 0.99) |
| 60 days | 0.87 (0.69; 1.08) | 0.88 (0.70; 1.10) | 0.81 (0.67; 0.98) | 0.82 (0.67; 0.99) |
| 90 days | 0.81 (0.66; 1.00) | 0.82 (0.66; 1.01) | 0.82 (0.67; 0.99) | 0.83 (0.68; 1.02) |
| Fourth^d^ |  |  |  |  |
| 30 days | 0.85 (0.61; 1.19) | 0.85 (0.61; 1.20) | 0.97 (0.77; 1.23) | 0.98 (0.77; 1.24) |
| 60 days | 0.86 (0.64; 1.15) | 0.85 (0.63; 1.15) | 0.98 (0.77; 1.25) | 0.98 (0.77; 1.25) |
| 90 days | 0.83 (0.63; 1.09) | 0.80 (0.60; 1.05) | 0.97 (0.75; 1.25) | 0.92 (0.71; 1.18) |
| Cox regression for all three outcomes. | | | | |
| Additional adjustment with year of hospitalization, the main discharge diagnosis, and exposure time. | | | | |
| a) 30 days: User: 713, Non-user: 6,332; 60 days: User: 681, Non-user: 5,828; 90 days: User: 632, Non-user: 5,150. | | | | |
| b) 30 days: User: 696, Non-user: 4,297; 60 days: User: 644, Non-user: 4,012; 90 days: User: 580, Non-user: 3,543. | | | | |
| c) 30 days: User: 473, Non-user: 3,044; 60 days: User: 429, Non-user: 2,701; 90 days: User: 370, Non-user: 2,261. | | | | |
| d) 30 days: User: 293, Non-user: 2,144; 60 days: User: 272, Non-user: 1,933; 90 days: User: 233, Non-user: 1,666. | | | | |
